# Sulfated Alginate as an Effective Polymer Binder for High-Voltage LiNi_0.5_Mn_1.5_O_4_ Electrodes in Lithium-Ion Batteries

**DOI:** 10.1021/acsami.2c11695

**Published:** 2022-11-09

**Authors:** Asako Oishi, Ryoichi Tatara, Eiichi Togo, Hiroshi Inoue, Satoshi Yasuno, Shinichi Komaba

**Affiliations:** † Department of Applied Chemistry, Tokyo University of Science, 1-3 Kagurazaka, Shinjuku, Tokyo 162-8601, Japan; ‡ Tosoh Corp., 1-8 Kasumi, Yokkaichi-Shi, Mie 510-8540, Japan; ¶ Japan Synchrotron Radiation Research Institute, 1-1-1 Kouto, Sayo-gun, Hyogo 679-5198, Japan

**Keywords:** lithium-ion battery, high-voltage spinel cathode, LiNi_0.5_Mn_1.5_O_4_, alginate, water-soluble binder

## Abstract

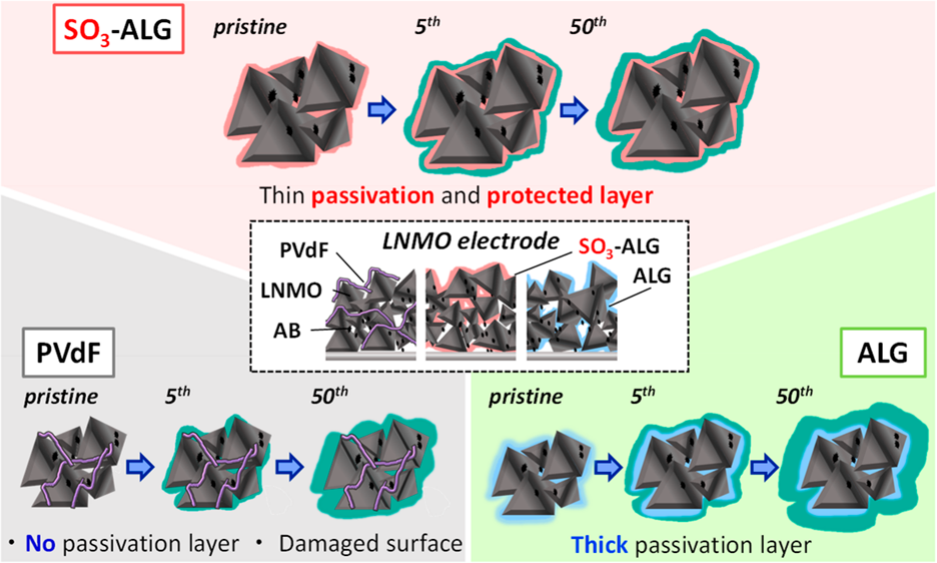

Although the increasing demand for high-energy-density lithium-ion batteries (LIBs) has inspired extensive research on high-voltage cathode materials, such as LiNi_0.5_Mn_1.5_O_4_ (LNMO), their commercialization is hindered by problems associated with the decomposition of common carbonate solvent–based electrolytes at elevated voltages. To address these problems, we prepared high-voltage LNMO composite electrodes using five polymer binders (two sulfated and two nonsulfated alginate binders and a poly(vinylidene fluoride) conventional binder) and compared their electrochemical performances at ∼5 V vs Li/Li^+^. The effects of binder type on electrode performance were probed by analyzing cycled electrodes using soft/hard X-ray photoelectron spectroscopy and scanning transmission electron microscopy. The best-performing sulfated binder, sulfated alginate, uniformly covers the surface of LNMO and increased its affinity for the electrolyte. The electrolyte decomposition products generated in the initial charge–discharge cycle on the alginate-covered electrode participated in the formation of a protective passivation layer that suppressed further decomposition during subsequent cycles, resulting in enhanced cycling and rate performances. The results of this study provide a basis for the cost-effective and technically undemanding fabrication of high-energy-density LIBs.

## Introduction

Despite their widespread use and diverse applications, lithium-ion batteries (LIBs) cannot fully satisfy the demands of the present-day society because of their insufficient energy density.^1^ Given that energy density can be increased by elevating the operating voltage,^2^ LiNi_0.5_Mn_1.5_O_4_ (LNMO) spinel has attracted significant attention as an LIB cathode material due to its high operating voltage (∼4.7 V vs Li/Li^+^) and higher theoretical energy density (∼1.2 times that of the commonly used LiCoO_2_).^3−5^ However, the practical application of LNMO is hindered by the tendency of carbonate-based electrolytes (e.g., LiPF_6_ with ethylene carbonate (EC) and dimethyl carbonate (DMC)) to undergo oxidative decomposition at voltages above 4.3 V during charge–discharge.^6^ Specifically, the deposition of decomposition products on LNMO and the leaching of transition metals from the active material by the generation of HF^7,8^ degrade the cycling and rate performances.^9^

The above problems can be mitigated in many ways. For example, severe electrolyte decomposition can be avoided by the use of additives,^10,11^ while metal oxide (e.g., CuO, ZnO, Al_2_O_3_) surface coatings and carbon materials can stabilize the electrode/electrolyte interface and suppress side reactions on the electrode surface to improve cycling performance.^12−15^ However, these strategies entail certain drawbacks, such as rate performance deterioration, high cost, and the need for complicated production processes.

In view of the current situation, we focus on binder optimization as a simpler and cheaper strategy for combating battery deterioration.^16−19^ Polymer binders should be mechanically stable, adhesive, and flexible to provide stiffness to the composite electrode, as well as having high oxidative/reductive stability. It is also required to have affinity for the electrolyte to be swelled to support ion conduction in the composite electrode but be insoluble to maintain electrode structure. In addition, polymer binders should be inexpensive and the production process should be efficient and environmentally friendly. Poly(vinylidene fluoride) (PVdF), which is widely used as a binder for practical LIB cathodes, must be dissolved in toxic *N*-methyl-2-pyrrolidone (NMP)^20^ and does not suppress electrolyte decomposition on LNMO or the leaching of transition metals from the spinel. Consequently, water-soluble binders are highly sought as green and cheap alternatives to PVdF.^21,22^ To date, we have investigated acrylic rubber and styrene–butadiene rubber/sodium carboxymethyl cellulose (Na-CMC) as water-soluble binders and demonstrated that the corresponding LIBs exhibit improved electrochemical performance.^23,24^ Water-soluble binders, such as Na-CMC,^25^ poly(vinyl alcohol),^26^ and lithium polyacrylate,^27^ have also been applied to LNMO cathodes, although further binder optimization is required. One important characteristic of the above polymer binders is the ability to form a passivation film at the electrode/electrolyte interface, which protects the surface of active materials to be decomposed during continuous cycling experiment, where the electrodes were exposed in highly oxidative/reductive condition. Further development of these “functional” binders is crucial to improve the performance of high energy density rechargeable batteries.

Alginate, a readily available natural polysaccharide extracted from brown algae that contains 1 → 4 linked β-d-mannuronic acid and α-l-guluronic acid residues, is significantly cheaper (8 vs 3 USD kg^–1^, respectively) and stiffer (0.6 vs 4.3 GPa, respectively) than PVdF.^28^ Its greater stiffness is attributed to the presence of hydroxy and carboxy groups in the alginate structure, which can improve the cycling performance of Si electrode in Li cell by suppressing electrode expansion.^29^ The cycling performance of cathode materials, such as LiMn_2_O_4_ and LiNi_0.5_Mn_1.5_O_4_, can also be improved by exploiting the ability of alginate to form hydrogels with di- or multivalent metal ions, such as Mn^2+^, thus solving the problematic leaching.^30−33^ However, the impact of electrolyte decomposition on the extent of Mn^2+^ leaching in cells with alginate binders has not been extensively investigated. In addition, although the replacement of PVdF by lithium dextran sulfate was reported to suppress electrolyte decomposition,^34^ the effects of binder sulfation on the electrochemical properties and electrode surface conditions of the corresponding cells are not fully understood. To bridge this gap, we newly synthesized sulfated alginates and examined the electrochemical properties of LNMO composite electrodes prepared using sulfated and nonsulfated alginate as binders. We found that the former effectively suppresses electrolyte decomposition at high voltages, resulting in a higher reversibility of LNMO electrode and superior passivation.

## Experimental Section

### Materials

**Figure 1:**
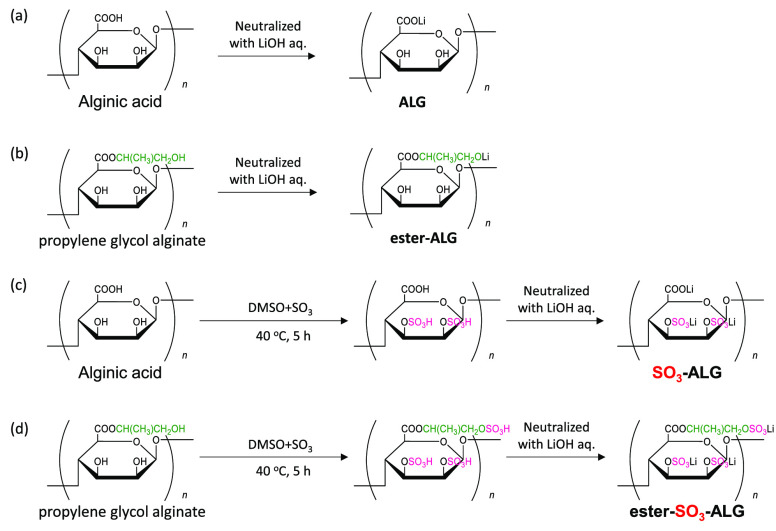
Syntheses and structures of alginate binders: (a) ALG, (b) SO_3_-ALG, (c) ester-ALG, and (d) ester- SO_3_-ALG.

Alginate binders (Figure 1) were prepared and supplied by TOSOH Corp. Lithium alginate (ALG) was prepared by neutralizing alginic acid (KIMICA Acid SA, KIMICA Co.) with a stoichiometric amount of LiOH. Sulfated ALG (SO_3_-ALG) and sulfated propylene glycol alginate (ester-SO_3_-ALG) were prepared by the partial sulfation of ALG and propylene glycol alginate (ester-ALG; KIMILOID HV, KIMICA Co.) as follows. A complex of SO_3_ with pyridine (90%, Fujifilm Wako Pure Chemical Co.) was added to a solution of ALG or ester-ALG in dimethyl sulfoxide, and the mixture was stirred at 40 °C for 5 h under N_2_. LNMO, which features Ni/Mn ordering with a *P*4_3_32 space group, was synthesized using a solid-state method. Li_2_CO_3_ (>99.0%, Kanto Chemical Co., Ltd.), Mn_2_O_3_, and NiCO_3_·2Ni(OH)_2_·4H_2_O (95.0%, Fujifilm Wako Pure Chemical Co.) were mixed in stoichiometric amounts and ball-milled for 12 h. Mn_2_O_3_ was prepared by calcining MnCO_3_ (Kishida Chemical Co., Ltd.) at 700 °C. The mixture was pelletized, and the pellets were calcined at 600 °C for 10 h in air, annealed at 900 °C for 12 h in air, and finally annealed at 750 °C for 48 h in an O_2_ atmosphere.^35^ Acetylene black (AB; Denka Black Li-400, Denka Co., Ltd.), graphite (SNO3, SEC carbon, particle size = 3 μm), PVdF (Polysciences Co.), Na-CMC (CMC#2200, Daicel Fine Chem, Ltd., substitution degree = 0.8–1.2), NMP (>99.0%, Kanto Chemical Co., Ltd.), and 1.0 M LiPF_6_ in 1:1 (v/v) EC/DMC (battery grade, Kishida Chemical Co., Ltd.) were used as received. Deionized water was prepared by purelite PRA-0015 (Organo).

### Measurements

Electrodes were prepared by thoroughly mixing LNMO/AB/binder in mass ratio of 80:10:10 (cathode) and a graphite/Na-CMC in mass ratio of 95:5 (anode). The corresponding mixtures were homogenized in NMP (PVdF binder) or in deionized water (alginate binders) with a planetary mixer (ARE-310, Thinky). The resulting slurries were cast on Al foil (thickness = 20 μm, Hosen Co., rough surface) and Cu foil (thickness = 20 μm, Hosen Co., mirror finish) to prepare the cathode and anode, respectively. For electrodes with alginate binders, the Al foil was pretreated with UV/ozone (SSP16–110, Sen Light) to increase surface hydrophilicity. The electrodes were dried at 80 °C in ambient air and then vacuum-dried at 100 °C. The mass loading of LNMO ranged from 2.3 to 3.5 mg cm^–2^. Electrodes with PVdF were pressed to 75% thickness using a roll press, whereas electrodes with alginate binders were not pressed. The resulting composite sheets were cut into 10 mm-diameter disks for battery testing. Cells containing electrodes with alginate binders were assembled using vacuum impregnation. Galvanostatic charge–discharge tests were conducted using R2032-type coin cells with Al caps (Hosen Co.) assembled in an Ar-filled glovebox (Miwa Manufacturing Co., Ltd.). Li-metal foil (Honjo Chemical Co.) was used as a counter electrode for half-cell tests (LNMO//Li), while full-cell tests were conducted using graphite as a counter electrode (LNMO//graphite) with 5%–15% excess capacity loaded on the graphite side to avoid Li plating at graphite. Porous polyolefin sheets (Toray Ind., Inc.) were used as separators. Electrolytes were vacuum-impregnated into composite electrode during cell assembly. Galvanostatic cycling was performed in at 25 ± 1 °C within a voltage range of 3.5–5.0 V (half cells) or 3.5–4.9 V (full cells) at a current rate of 20 mA g^–1^. Electrochemical impedance spectroscopy (EIS; VMP3, Biologic) measurements were performed in a three-electrode setup (Toyo Systems; reference electrode = Ni mesh with deposited Li, counter electrode = Li-metal foil),^36−38^ after charging to 50 mAh g^–1^, at an amplitude of 5 mV within a frequency range of 100 kHz to 10 mHz.

The structural change of LNMO electrodes during battery cycling was probed by X-ray diffractometry using Ni-filtered Cu Kα radiation operating at 45 mA and 40 kV (Smartlab, Rigaku Corp.) in Bragg–Brentano geometry with a 1D silicon strip detector (D/tex Ultra 250, Rigaku Corp.). A focused ion beam (FIB; JIB-4501, JEOL) was used to cut cycled LNMO electrodes across the contact region for further surface analysis. Transmission electron microscopy (TEM; JEM-ARM200F, JEOL) imaging of cycled electrodes was performed at an acceleration voltage of 200 kV for samples processed by FIB into thin slices (10 μm × 10 μm × several tens of nanometers) and placed on a Cu grid. Attenuated total reflection-Fourier transform infrared (ATR-FTIR) spectroscopy (Bruker ALPHA 2) was used to probe the chemical structure of alginate binder films obtained by adding deionized water to LiOH-neutralized solutions of binders and drying at 80 °C in air. The surface components and characteristics of fresh and cycled LNMO electrodes were probed by soft X-ray photoelectron spectroscopy (SOXPES) and hard X-ray photoelectron spectroscopy (HAXPES). For SOXPES, which is known as lab-scale conventional XPS, measurements were performed using a JPS-9010MC instrument (JEOL, Ltd.) equipped with a nonmonochromatic Mg Kα X-ray source (1253.6 eV). HAXPES measurements were conducted at a high excitation energy of 7938.9 eV using an R-4000 (Scienta Omicron AB) photoelectron energy analyzer at the BL46XU beamline of the SPring-8 facility, Hyogo, Japan. The binding energies for both HAXPES and SOXPES were calibrated using the O 1s peak of the LNMO lattice oxygen at 529.7 eV as a reference. The integrated peak intensities were normalized by the O 1s peak of the LNMO lattice oxygen after baseline correction. Cycled electrodes were carefully taken out from the coin cell, washed with DMC (>99.5%, battery grade, Kishida Chemical Co., Ltd.), and dried at room temperature under ambient pressure in a glovebox. The samples were transferred to the HAXPES chamber using a transfer vessel without exposure to air. Samples for SOXPES measurement were sealed in an aluminum laminate package with a heat sealer inside the glovebox for transport, before being opened and quickly placed into the chamber, minimizing air exposure.

## Results and Discussion

### Properties of Alginate Binders

The fundamental properties of alginate binders are listed in Table 1. The sulfation efficiency of hydroxy groups (number of sulfated hydroxy groups divided by the total number of hydroxy groups in Figure 1c,d) in SO_3_-ALG and ester-SO_3_-ALG is close to 30%. All binders are confirmed to be almost insoluble in the EC/DMC solvent (Table 1). The corresponding electrodes are therefore concluded to be stable in contact with the electrolyte employed. Sulfated binders (SO_3_-ALG and ester-SO_3_-ALG) show greater solvent uptake than nonsulfated (ALG and ester-ALG) ones, which is ascribed to the high polarity of the sulfate group and the resulting increase in electrolyte affinity, as previously reported for other polar groups.^39^

**Table 1:** Selected Properties of Tested Binders in This Study

binder		ALG	SO_3_-ALG	ester-ALG	ester-SO_3_-ALG	PVdF^45^
sulfur content (wt %)^a^		0.3	10.1	0.1	9.2	
sulfation efficiency (%)^b^		0	37	0	29	
ion-exchange capacity (meq g^–1^)^c^		0	3.2	0	2.9	
molecular weight (×10^4^ Da)^d^	*M*_n_	3	2.5	12	10	
	*M*_w_	6.2	4.8	44	31	
polydispersity index^d^		2.1	1.9	3.7	3	
solvent uptake^e^/%		17	28	29	39	20
solubility^f^/%		99	96	98	98	97

a Determined by elemental analysis.

b Calculation detail can be found in the Supporting Information.

c The reaction product was dissolved in water, and the solution was dropwisely added to 1 M aqueous HCl to precipitate SO_3_-ALG and ester-SO_3_-ALG. The precipitate was collected by filtration, washed with ethanol, dried, and immersed in saturated NaCl solution for 2 h. The aqueous solution was separated, and the HCl produced was quantified by titration with NaOH/phenolphthalein to determine the cation-exchange capacity.

d Determined by gel permeation chromatography.

e Compare the masses of the film between before (*w*_0_) and after (*w*_1_) immersion. The mass increment corresponds to the solvent uptake: solvent uptake (%) = [(*w*_1_ – *w*_0_)/*w*_0_] × 100.^45^

f The film was dried again at 80 °C in a vacuum to remove EC/DMC. The resulting mass (*w*_2_) was used to calculate the solubility of the binder: solubility (%) = [(*w*_0_ – *w*_2_)/*w*_0_] × 100.^45^

**Figure 2:**
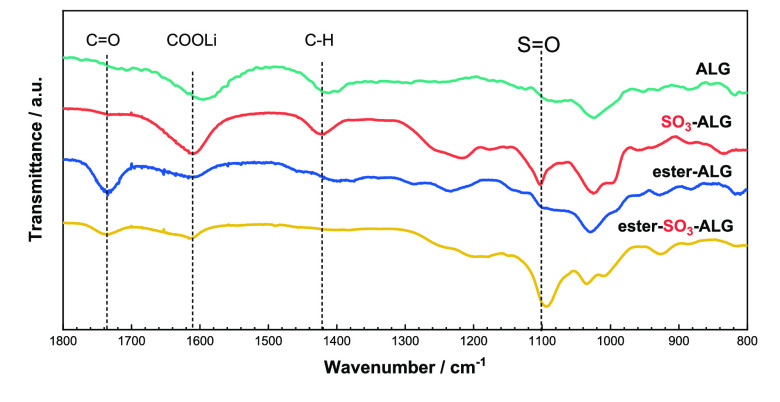
FTIR spectra of alginate binders (ALG, SO_3_-ALG, ester-ALG, and ester-SO_3_-ALG) used in this study. Pure polymer film was used for the measurements with diamond ATR prism.

Figure 2 presents the ATR-FTIR spectra of alginate binders. The spectra of ester-ALG and ester-SO_3_-ALG feature an ester C=O stretch at ∼1750 cm^–1^,^40^ while the spectra of all binders feature an asymmetric stretch of —COOLi groups at ∼1600 cm^–1^,^41−43^ and the spectra of SO_3_-ALG and ester-SO_3_-ALG feature a sulfate S=O stretch at ∼1100 cm^–1^.^44^ These results confirm the presence of sulfate groups in SO_3_-ALG and ester-SO_3_-ALG.

### Performance of LNMO Electrodes with Alginate Binders

**Figure 3:**
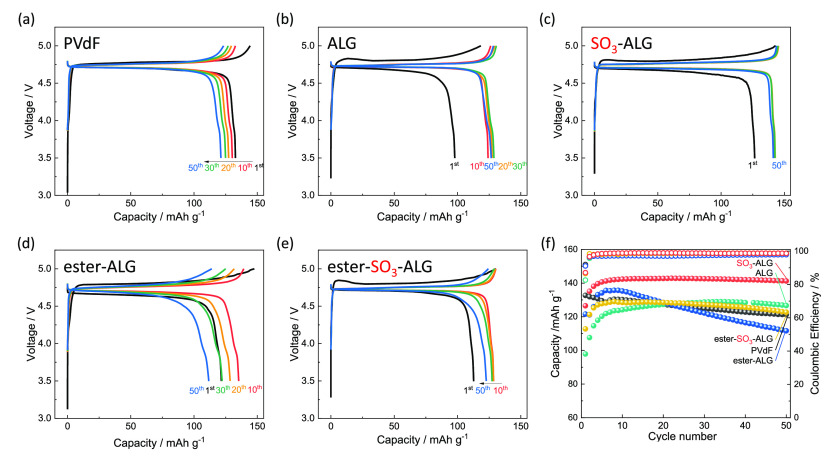
Charge–discharge curves of LNMO//Li half cells with (a) PVdF, (b) ALG, (c) SO_3_-ALG, (d) ester-ALG, and (e) ester-SO_3_-ALG binders. (f) Variation of the capacities and Coulombic efficiencies of LNMO//Li half cells with PVdF and alginate binders. The cells were cycled in the voltage range 3.5–5.0 V at 20 mA g^–1^ at 25 °C using 1 M LiPF_6_ in EC/DMC as an electrolyte.

The charge–discharge curves of LNMO//Li half cells with different binders in Figure 3a–e reveal a distinct plateau attributed to the Ni^2+/4+^ redox couple near 4.7 V in all cases.^35^ In general, two polymorphs of LNMO are known: Ni/Mn-ordered LNMO with a space group of *P*4_3_32 and Ni/Mn disordered one with *Fd*-3*m*. While *Fd*-3*m* shows superior cyclability, *P*4_3_32 provides a higher energy density due to its slightly higher redox potential.^35^ The *P*4_3_32 space group with a lower cyclability was selected in this study to demonstrate the effect of the binder polymer structure. In LNMO (*P*4_3_32), Ni, Mn, and Li atoms are regularly located at the 4b, 12d, and 8c sites, respectively. The Li^+^ ions in 8c sites diffuse in the crystal structure, with one-third of these ions migrating through the proximal 4a sites and two-thirds migrating through the distal 12d sites.^46^ The theoretical capacity of LNMO is 147 mAh g^–1^, which corresponds to the oxidation of Ni^2+^ to Ni^4+^ at ∼4.7 V vs Li^+^/Li.^46^Figure 3 shows that the discharge capacity of the PVdF electrode decreases monotonically with an increasing number of cycles, whereas that of alginate electrodes increases over initial 10 or more cycles, possibly because the discharge capacity in the initial cycle is small owing to overvoltage caused by slow electrolyte penetration inside the porous LNMO composite electrode. The initial discharge capacity and Coulombic efficiency was decreased if the electrolyte was not vacuum impregnated into composite electrode during cell assembly (Figure S1). In addition, a lower binder content in the composite electrode decreases the cyclability (Figure S2), indicating that 10 wt % binder content is optimized value. A marginally low initial Coulombic efficiency may originate from slightly low oxidative stability in alginate-based binder compared with fluorinated PVdF (Figure S3). In the case of SO_3_-ALG, a discharge capacity of 140 mAh g^–1^ is reached at the 10th cycle, and the shape of the charge–discharge curve hardly changes until the 50th cycle, with no discharge capacity degradation observed. Figure 3f shows the effects of cycling on discharge capacity and Coulombic efficiency of LNMO electrodes, illustrating that the capacity of the PVdF electrode decreases from 132 to 121 mAh g^–1^ after 50 cycles. Notably, the capacities of the alginate electrodes are lower than that of the PVdF electrode after the first discharge but subsequently increase and stabilize at values exceeding that of the PVdF electrode. The ALG and ester-ALG electrodes show a decrease in discharge capacity over 50 cycles, whereas the SO_3_-ALG and ester-SO_3_-ALG electrodes show almost no capacity decay over 50 cycles. In the case of SO_3_-ALG, the capacity of 140 mAh g^–1^ obtained after 50 cycles is close to the theoretical value of 147 mAh g^–1^. The increase in discharge capacity during the first 10 cycles observed for electrodes with alginate binders is accompanied by a decrease in polarization and is therefore not due to initial lithium loss. As described later, the thin layer of alginate binders cover the active material surface, thus reducing interfacial resistance by facilitating the penetration of electrolyte and the formation of a passivation layer over ∼10 cycles.^25,47^ The average Coulombic efficiency during 50 cycles decreases in the order SO_3_-ALG (98.5%) > ester-SO_3_-ALG (98.4%) > ALG (98.0%) > PVdF (98.0%) > ester-ALG (97.2%). The higher efficiency of electrodes can be achieved with sulfated binders, which is attributed to the ability of these binders to promote the formation of a uniform and stable protective passivation layer during the initial cycle. Thus, the introduction of sulfate groups improves cycling performance.

**Figure 4:**
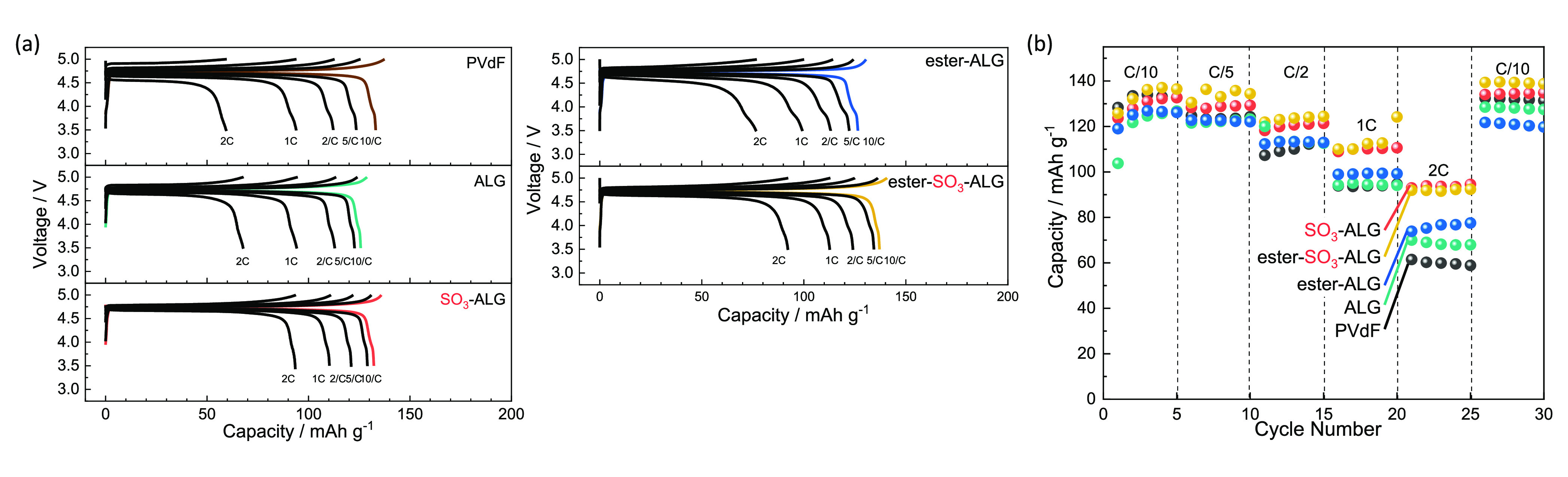
Rate capabilities of LNMO electrodes with alginate binders. (a) Charge–discharge curves of LNMO//Li half cells with PVdF and alginate binders. (b) Rate capability at different rates, increased from C/10 to 2C.

Figure 4 presents the rate capabilities of LNMO electrodes with different binders. At C/10 (0.06 mA cm^–2^), the discharge capacity is close to 130 mAh g^–1^ regardless of the binder, although capacity differences among the binders become more pronounced at higher current densities, where capacities of 61, 70, 73, 93, and 92 mAh g^–1^ are obtained at 2 C for PVdF, ALG, ester-ALG, SO_3_-ALG, and ester-SO_3_-ALG, respectively. The higher values obtained for SO_3_-ALG and ester-SO_3_-ALG are attributed to the higher polarities of these binders. As shown in Table 1, ion-conduction paths are formed in the SO_3_-ALG and ester-SO_3_-ALG electrodes because of their improved electrolyte affinity. Also, the diffusion of Li^+^ ions in the entire composite electrode is enhanced by the SO_3_-binders, which results in a large discharge capacity at a high rate. Therefore, we compared the behaviors of the PVdF, ALG, and SO_3_-ALG electrodes to investigate the effect of sulfation in greater detail.^48^

**Figure 5:**
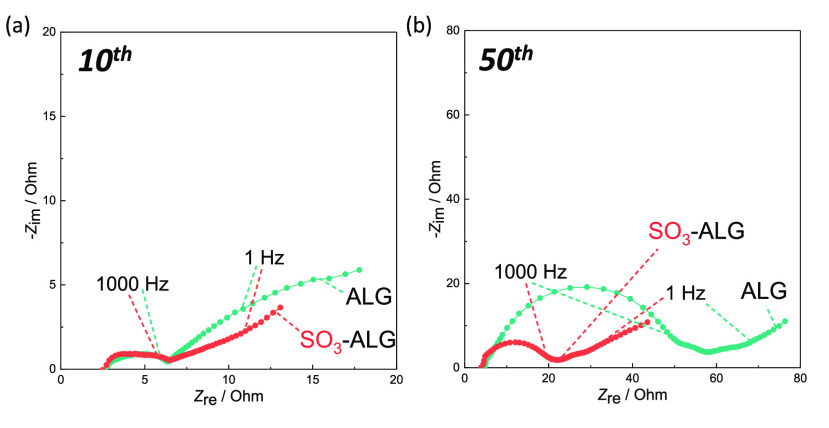
Nyquist plots of LNMO electrodes with ALG and SO_3_-ALG binders recorded at 50 mAh g^–1^ charging at the (a) 10th and (b) 50th cycles.

To elucidate the origin of the improved cycling and rate performances observed for sulfated binders, we performed EIS measurements and determined the interfacial resistances on the cathode side. LNMO electrode with PVDF binder is known to show increasing interfacial resistance.^24,55^Figure 5 shows the Nyquist plots obtained after 10 and 50 cycles at 50 mAh g^–1^ charging. As the assignment of Nyquist plots for composite electrodes is complicated even in the case of three-electrode measurements,^36,49−53^ we only discuss the size of the entire capacitive semicircle as an indicator of interfacial resistance. The ALG and SO_3_-ALG electrodes show semicircles of a similar size at the 10th cycle, whereas the latter electrode shows a smaller semicircle at the 50th cycle. Thus, the introduction of sulfate groups appears to suppress the increase in interfacial resistance upon cycling, possibly by favoring the formation of a stable protective layer on the initial cycle, thus suppressing the deposition of electrolyte decomposition products and/or the surface degradation of LNMO in subsequent cycles.

As the superior cycling performance of electrodes with alginate binders, especially in the case of SO_3_-ALG, is due to low interfacial resistance, we analyzed the surfaces of cycled electrodes to find out the origin of this resistance. Note that the bulk crystal structure is almost fully retained after repeated charge–discharge reactions (Figure S4).

**Figure 6:**
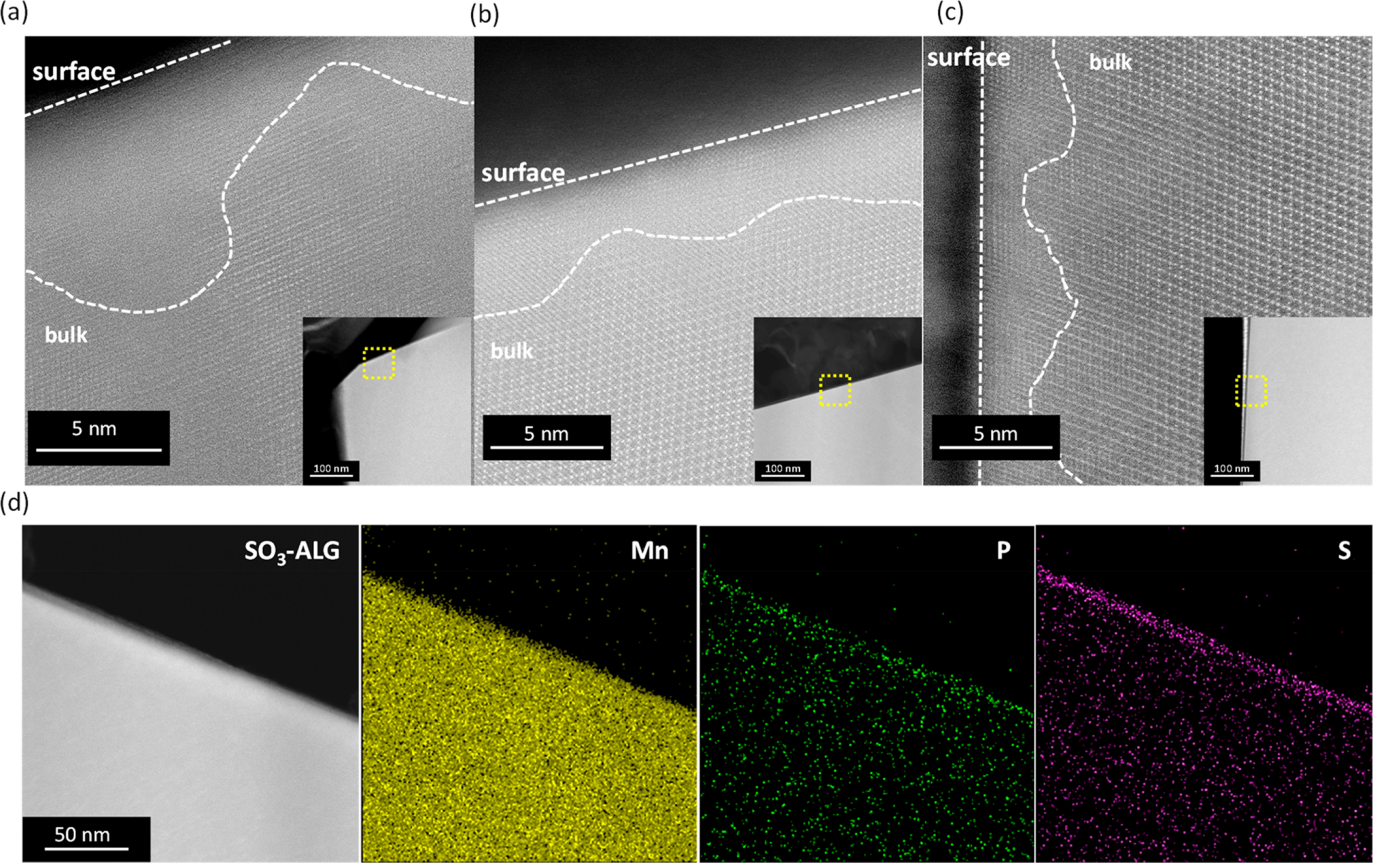
FIB-STEM images of LNMO particles in 50-fold cycled electrodes with (a) PVdF, (b) ALG, and (c) SO_3_-ALG. (d) Mn, P, and S EDS mappings of the 50-fold cycled SO_3_-ALG electrode.

Figure 6 shows representative scanning transmission electron microscopy (STEM) images of 50 cycled electrodes with different binders and energy-dispersive X-ray spectroscopy (EDS) elemental mappings of the cycled SO_3_-ALG electrode. Both methodologies reveal the lattice fringes on the surface of the LNMO particles to be less distinct than those on the bulk surface, indicating structural degradation from the electrode/electrolyte interface. The observed behavior is ascribed to the reaction of LiPF_6_ with a small amount of water in the electrolyte to form HF (LiPF_6_ (sol.) + H_2_O → POF_3_ (sol.) + LiF (s) + 2HF (sol.), PF_5_ (sol.) + H_2_O → POF_3_ (sol.) + 2HF (sol.)), which induces the leaching of Mn^2+^ and Ni^2+^ from LNMO and causes the surface degradation of LNMO particles.^7,24,54^ The thickness of the degraded area exceeds 10 nm for the PVdF electrode but is close to 5 nm for the ALG and SO_3_-ALG electrodes. Thus, electrodes with alginate binders better maintain their structure near the surface. The analysis of surface-layer components by STEM coupled with EDS reveals that, in the case of the SO_3_-ALG electrode, which exhibits the best cycling performance (Figure 3), LNMO is uniformly coated with a sulfur-containing layer, which is 10 nm thick. The absence of sulfur species in the 1.0 M LiPF_6_/EC-DMC electrolyte suggests that the SO_3_-ALG binder uniformly covers the surface of the LNMO particles and acts as a protective layer. The signals from electrolyte decomposition-derived phosphorus species are pronounced on the LNMO particle surface. As phosphorus is contained only in the electrolyte, the surface of the LNMO particles is concluded to be covered by both the binder and electrolyte decomposition products. This conclusion is consistent with previous reports on the use of high-solvent-uptake binders, wherein the binders covering the cathode surface were shown to decompose and form a passive layer by anodic decomposition of the electrolyte.^24,45,55^

**Figure 7:**
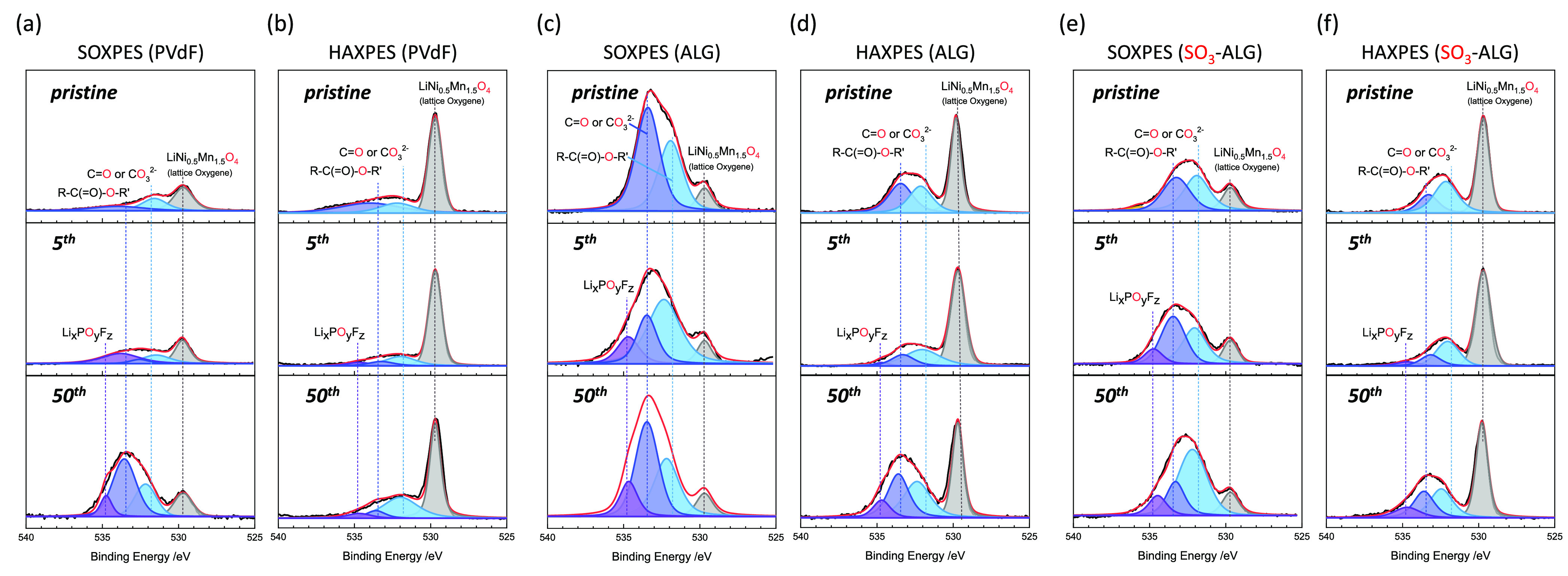
O 1s photoelectron spectra of LNMO electrodes with different binders after zero, five, and 50 cycles. (a) SOXPES and (b) HAXPES of electrodes with PVdF. (c) SOXPES and (d) HAXPES of electrodes with ALG. (e) SOXPES and (f) HAXPES of electrodes with SO_3_-ALG. The cells were cycled in the voltage range 3.5–5.0 V at 20 mA g^–1^ at 25 °C using 1 M LiPF_6_ in EC/DMC as an electrolyte

The surface layer on LNMO was further examined by the SOXPES and HAXPES of electrodes after zero (pristine electrode), 5, and 50 cycles. These techniques provide information at depths of several to several tens of nanometers from the surface, respectively.^56^ For ease of comparison, each spectrum was processed using binding energy calibration and peak normalization with respect to the O 1s signal of lattice oxygen at 529.7 eV.^57^Figure 7 shows the acquired O 1s photoelectron spectra of the LNMO electrodes. In the SOXPES profile of PVdF, the lattice oxygen peaks (529.7 eV)^57^ of pristine and five cycled electrodes are stronger than (i) the C=O (531.8 eV)^58^ and R—C(=O)—OR (533.3 eV)^59^ peaks attributed to the products of solvent decomposition and (ii) the peak of Li_*x*_PO_*y*_F_*z*_ (534.5 eV)^58^ produced by the electrolytic reaction of POF_3_ (POF_3_ + 2*xe*^–^ + 2*x*Li^+^ → *x*LiF + Li_*x*_PF_3–*x*_O), which can be formed by the reaction of LiPF_6_ with traces of water in the electrolyte (LiPF_6_ + H_2_O → LiF + 2HF + POF_3_). However, after 50 cycles, the lattice oxygen peak becomes weaker than the C=O, R—C(=O)—OR, and Li_*x*_PO_*y*_F_*z*_ peaks. In particular, the C=O and R—C(=O)—OR peaks associated with the products of solvent decomposition gain significantly in intensity between the fifth and 50th cycles. Therefore, EC and DMC decomposition products are formed on the top surface of the deposit on LNMO,^60^ which suggests that contact between the electrolyte and LNMO is not interrupted and that the decomposition reaction proceeds continuously upon cycling. The fact that the lattice oxygen peak is still observed after 50 cycles indicates that the thickness of the surface layer is less than the SOXPES analysis depth or imperfect coverage of the surface deposits. In the HAXPES profiles, the lattice oxygen peak is stronger than other peaks regardless of the number of cycles. This behavior is the result of the greater analysis depth of HAXPES, which provides more information about the active material than the thin overlayer. However, even though the peaks derived from the film are weak, the signals from the electrolyte decomposition products (e.g., C=O and R—C(=O)—OR) gain intensity as the number of cycles increases, in agreement with the SOXPES results. Note that the spectrum of the electrode soaked in the electrolyte is similar to that of the pristine electrode (Figure S5).

As shown in Figure 7c for ALG, the C=O and R—C(=O)—OR SOXPES peaks of the pristine electrode are much stronger than the corresponding lattice oxygen peaks, which we attribute to the greater coverage of LNMO by ALG than by PVdF, where those peaks were weaker than lattice oxygen peak. In addition, the peaks of solvent decomposition products (C=O, R—C(=O)—OR)^60,61^ increase slightly in intensity between the fifth and 50th cycles, and a Li_*x*_PO_*y*_F_*z*_ peak indicative of electrolyte decomposition emerges that was not observed for the pristine electrode (O 1s spectra in Figure 7). However, the relative increase in intensity of the decomposition products is lower than that observed for PVdF by comparing Figure 7a and c. In the HAXPES measurements (Figure 7d), a lattice oxygen peak like that of PVdF is clearly observed. These results suggest that ALG suppresses the continuous decomposition of electrolyte more effectively by covering LNMO with a 3–10 nm-thick layer that prevents direct contact with the electrolyte. In the SOXPES measurements of SO_3_-ALG (Figure 7e), the C=O and R—C(=O)—OR peaks are stronger than the lattice oxygen peak (as found for ALG), which reflects the presence of SO_3_-ALG coating layer on LNMO. The peak intensities of the products of electrolyte decomposition (C=O, R—C(=O)—OR, Li_*x*_PO_*y*_F_*z*_) do not change significantly between the fifth and 50th cycles, as also observed in the HAXPES measurements (Figure 7f).

The above results are consistent with those of STEM-EDS imaging and suggest that, for highly polar SO_3_-ALG with uniform coating properties, the electrolyte adsorbs on the active material surface, and the solvent and electrolyte decomposition products produced in the initial cycle are deposited and entrapped in the SO_3_-ALG thin layer on LNMO, leading to formation of a protective layer, which precludes direct contact between LNMO and the electrolyte and suppresses decomposition during successive cycles. The C 1s spectra shown in Figures S6 and S7 show that the peak intensities of the CH(COOH) and O—CH moieties produced by oxidative decomposition of EC and DMC are almost unchanged from the fifth to the 50th cycle when SO_3_-ALG is used. The trend agrees with the above discussion in the O 1s spectra.

**Figure 8:**
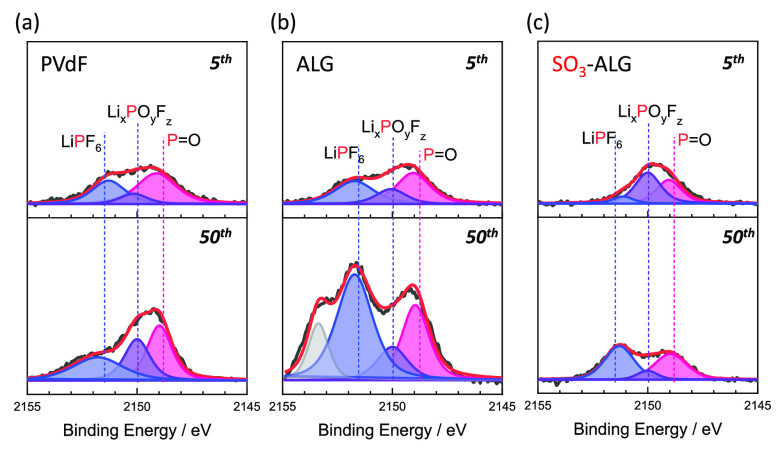
P 1s photoelectron spectra (HAXPES) of LNMO electrodes with (a) PVdF, (b) ALG, and (c) SO_3_-ALG binders, after five and 50 cycles. The cells were cycled in the voltage range 3.5–5.0 V at 20 mA g^–1^ and at 25 °C using 1 M LiPF_6_ in EC/DMC as an electrolyte.

Figure 8 shows the P 1s HAXPES profiles of LNMO electrodes after five and 50 cycles. After five cycles, the peak intensities of the Li_*x*_PO_*y*_F_*z*_ (2149.7 eV)^45^ and P=O (2148.5 eV)^62^ moieties produced through cycled with LiPF_6_ decomposition are similar to both alginate binders. This behavior is consistent with the fact that the variation in irreversible capacities (Figure S8) resembles until the fifth cycle. Thereafter, the increases in peak intensity of the electrolyte decomposition products imply growth of the electrolyte decomposition layer from five to 50 cycles for PVdF and ALG. However, the peak intensities of the electrolyte decomposition products remain almost unchanged from five to 50 cycles with SO_3_-ALG. Therefore, for PVdF and ALG, electrolyte decomposition progresses and the amount of material deposited increases with continued cycling. SO_3_-ALG coating is beneficial to promote the formation of a stable protective layer during the initial cycle(s), which hinders direct contact between LNMO and the electrolyte and suppresses electrolyte decomposition on subsequent cycles. This behavior is consistent with the trend observed in the O 1s (Figure 7) and F 1s (Figure S9) spectra and also with the fact that (unlike in the case of ALG) interfacial resistance does not increase when SO_3_-ALG is used (Figure 5). Since the rate capability is superior for the SO_3_-ALG (Figure 4), the protective layer should be good Li^+^ ion conductor, which is similar to SEI for Li-GIC.^63,64^ We further carried out self-discharge tests. After 10 charge–discharge cycles, the LNMO//Li half cells were fully charged, stored under open-circuit conditions for 7 days, and discharged for the 11th time. As seen in Figure S10 and Table S1, the SO_3_-ALG electrode shows a greater Coulombic efficiency, indicating a suppression of self-discharge compared to with the ALG one. This behavior is attributed to the fact that SO_3_-ALG exhibits the proper coating properties by forming the low-resistance passivating layer that suppresses electrolyte decomposition even at high voltages.^55^

**Figure 9:**
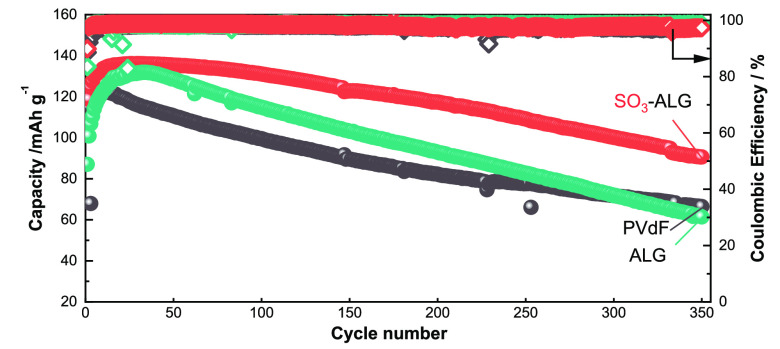
Long-term cycling performances and Coulombic efficiencies of LNMO//Li half cells with different binders. The cells were cycled in the voltage range 3.5–5.0 V at 20 mA g^–1^ at 25 °C using 1 M LiPF_6_ in EC/DMC as an electrolyte.

The foregoing XPS results indicate that the electrolyte decomposition products are accumulated with the number of cycles, because of the insufficient coverage of LNMO by the PVdF and ALG binders. On the other hand, the employment of SO_3_-ALG as binder is effective in forming a stable protective layer on the initial cycle, and decomposition of the electrolyte is suppressed during subsequent cycles. Thus, the introduction of sulfate groups is responsible for suppressing continuous electrolyte decomposition and improving electrochemical performance. Figure 9 shows the long-term cycling performances of different LNMO electrodes in Li cells. After 300 cycles, the PVdF and ALG electrodes show capacities of ∼70 mAh g^–1^, whereas the SO_3_-ALG electrode shows a capacity of >100 mAh g^–1^, indicative of much better cycling performance. Figure S11 shows the cycling performances of LNMO//graphite full cells, which accord with the half-cell results in that the SO_3_-ALG electrode has a higher discharge capacity and Coulombic efficiency than those of PVdF and ALG electrodes. This behavior is also attributed to the effective protection of the LNMO surface by the SO_3_-ALG derived passivation layer. In general, Mn dissolution from the spinel cathode occurred and the dissolved Mn^2+^ ions diffuse through the electrolyte and can be deposited as metallic Mn on the graphite negative electrode surface, causing capacity fading.^65^ However, Mn deposition is not observed on graphite electrodes with ALG-based binders in full cell, whereas a small Mn peak is observed for the graphite anode of the full cell with PVdF binder (Figure S12 and Table S2). Mn dissolution from LNMO severely reduces the capacity of the corresponding full cell during cycling, and the effective passivation of the LNMO surface also contributes to the suppression of transition metal dissolution.

**Figure 10:**
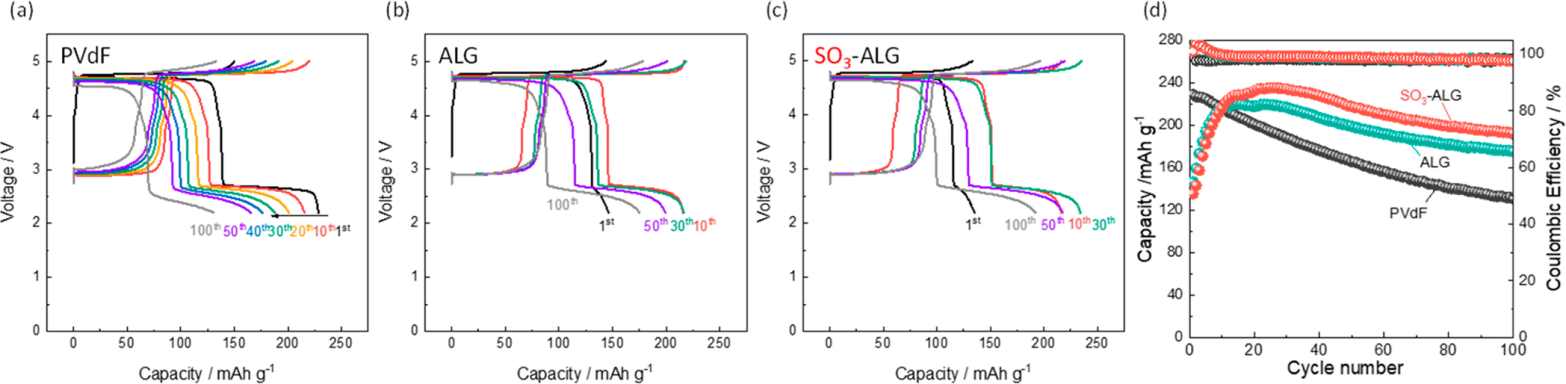
Charge–discharge curves of LNMO//Li half cells cycled in the voltage range 2.2–5.0 V with (a) PVdF, (b) ALG, and (c) SO_3_-ALG. (d) Effects of cycling on the capacities and Coulombic efficiencies of LNMO//Li half cells with PVdF and alginate binders. The cells were cycled at 20 mA g^–1^ and 25 °C using 1 M LiPF_6_ in EC/DMC as an electrolyte.

Finally, we present the results of charge–discharge tests conducted in the wider voltage range 2.2–5.0 V in Figure 10. Further discharging below 2.5 V provides extra capacity arising from the lithiation of Li_*x*_Ni_0.5_Mn_1.5_O_4_ (*x* ≥ 1). At the plateau near 2.5 V where 1 ≤ *x* ≤ 2, lithium participates in a two-phase reaction, wherein Li^+^ is inserted into the empty 16c octahedral sites accompanied by the reduction of Mn^4+^ to Mn^3+^.^66,67^ This phase transition is accompanied by anisotropic Jahn–Teller distortion and the elongation of the *c*-axis of the cubic lattice, leading to a structural change in the tetragonal phase.^68^ The PVdF-based electrode shows continuous capacity decay upon cycling, which is consistent with the cycling tests conducted in the voltage range 3.5–5.0 V (Figure 3). Conversely, the electrodes with alginate binders show a gradual increase in discharge capacity. This increase during the first 10 cycles derives from a similar overpotential, as mentioned in Figure 3. Figure 10 confirms that the SO_3_-ALG electrode shows a discharge capacity of 230 mAh g^–1^ with a mean discharge voltage of 3.9 V between 10th and 40th cycles. SO_3_-ALG demonstrates a higher capacity and Coulombic efficiency with better retention, providing an energy density of 897 Wh kg^–1^_positive_ at the 30th cycle. This value is greater than that of the common positive electrode material, LiNi_1/3_Mn_1/3_Co_1/3_O_2_ (∼650 Wh kg^–1^_positive_ at ≤4.4 V cycling).^69^ In general, discharging manganese spinel below 3 V will lead to fast capacity loss due to the serious Jahn–Teller distortion derived from Mn^3+^ with ∼10% volume change. This study proves that the above capacity decay can be improved by binder chemistry for the first time, where a similar effect was reported in our previous study of the glutamate binder in the Na_2/3_Ni_1/3_Mn_2/3_O_2_ positive electrode.^70^ Other cathode materials such as LiMn_2_O_4_ will also be examined in a future study.

## Conclusion

Alginate binders with sulfate groups were synthesized and applied to a high-voltage spinel LiNi_0.5_Mn_1.5_O_4_ electrode. The introduction of polar sulfate groups provided more stable cycling behavior and improved rate performance by binding more strongly to the electrode and assisting the adsorption of electrolyte. The best-performing binder, SO_3_-ALG, uniformly coated the electrode surface and promoted the formation of a protective passivating layer in the initial cycle that hindered the continuous degradation of the solvent and electrolyte at high voltage during subsequent cycles.

## Supporting Information Available

Discussion of calculation to obtain sulfation efficiency, figures of variation of the capacities and Coulombic efficiencies, cyclic voltammograms, XRD patterns, HAXPES profiles, SOXPES profiles, effects of cycling on the irreversible capacity of LiNi_0.5_Mn_1.5_O_4_//Li half cells, charge–discharge curves, cycling performances, and SEM-EDS profiles, and tables of 11th-cycle Coulombic efficiencies of LiNi_0.5_Mn_1.5_O_4_ electrodes with various binders obtained after self-discharge and surface atomic ratios estimated from the EDS profiles of graphite electrodes (PDF)

## Abbreviations Used

ALG: lithium alginate
ATR-FTIR: attenuated total reflection-Fourier transform infrared
DMC: dimethyl carbonate
EC: ethylene carbonate
EIS: electrochemical impedance spectroscopy
FIB: focused ion beam
HAXPES: hard X-ray photoelectron spectroscopy
LIB: lithium-ion battery
LNMO: LiNi_0.5_Mn_1.5_O_4_
Na-CMC: sodium carboxymethyl cellulose
NMP: *N*-methyl-2-pyrrolidone
SEM: scanning electron microscopy
STEM: scanning transmission electron microscopy
SOXPES: soft X-ray photoelectron spectroscopy
TEM: transmission electron microscopy
