# Supplementary material for: Sulfated Alginate as an Effective Polymer Binder for High-Voltage LiNi0.5Mn1.5O4 Electrodes in Lithium-Ion Batteries
Source: ACS Appl Mater Interfaces. 2022 Nov 9;14(46):51808–18. doi: 10.1021/acsami.2c11695 (PMC9706501; doi:10.1021/acsami.2c11695)
Supplement: Supplementary file 1 — am2c11695_si_001.pdf [file am2c11695_si_001.pdf]

Supporting Information:

Sulfated Alginate as an Effective Polymer Binder for High-Voltage $LiNi_{0.5}Mn_{1.5}O_4$ Electrodes in Lithium-Ion Batteries

Asako Oishi,[†] Ryoichi Tatara,[†] Eiichi Togo,[‡] Hiroshi Inoue,[‡] Satoshi Yasuno,[¶] Shinichi Komaba[†*]

[†]*Department of Applied Chemistry, Tokyo University of Science, 1-3 Kagurazaka, Shinjuku, Tokyo 162-8601, Japan*

[‡]*Tosoh Corp., 1-8 Kasumi, Yokkaichi-Shi, Mie 510-8540, Japan*

[¶]*Japan Synchrotron Radiation Research Institute, 1-1-1 Kouto, Sayo-gun, Hyogo, 679-5198, Japan*

**Email: komaba@rs.tus.ac.jp*

Calculation to obtain sulfation efficiency in Table 1:

Unit mass of ALG: 176.06 [g/mol]

Unit mass of ALG-SO_3 with x% sulfation efficiency: 176.06+160.13x [g/mol]

As we have relationship as follows:

$(64.13x/(176.06+160.13x)) \times 100$ = sulfur content [wt%, elemental analysis]

Through solving above equation for x, we obtain sulfation efficiency.

Unit mass of ester-ALG: 234.09 [g/mol]

Unit mass of ester-ALG-SO_3 with x% sulfation efficiency: 234.09+240.195x [g/mol]

As we have relationship as follows:

$(234.09x/(234.09+240.195x)) \times 100$ = sulfur content [wt%, elemental analysis]

Through solving above equation for x, we obtain sulfation efficiency.

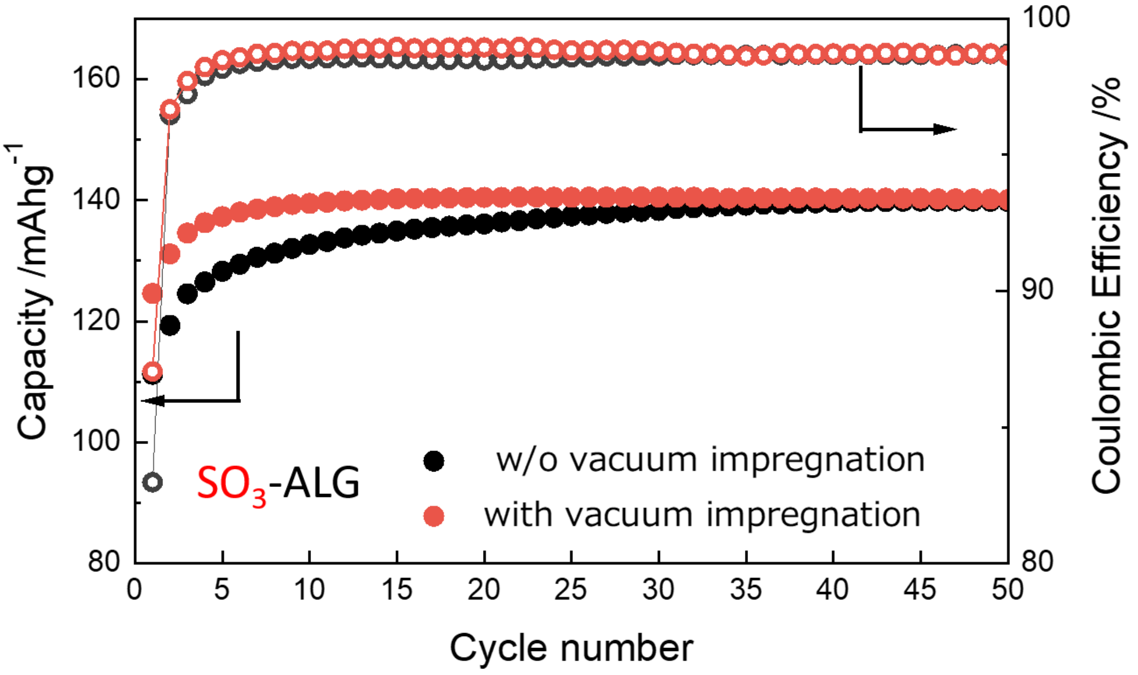

Figure S1. Variation of the capacities and Coulombic efficiencies of LNMO//Li half cells with SO_3-ALG binders, with and without electrolyte vacuum impregnation process. The cells were cycled in the voltage range of 3.5–5.0 V at 20 mA g^{-1} at 25 °C using 1 M $LiPF_6$ in EC/DMC as an electrolyte.

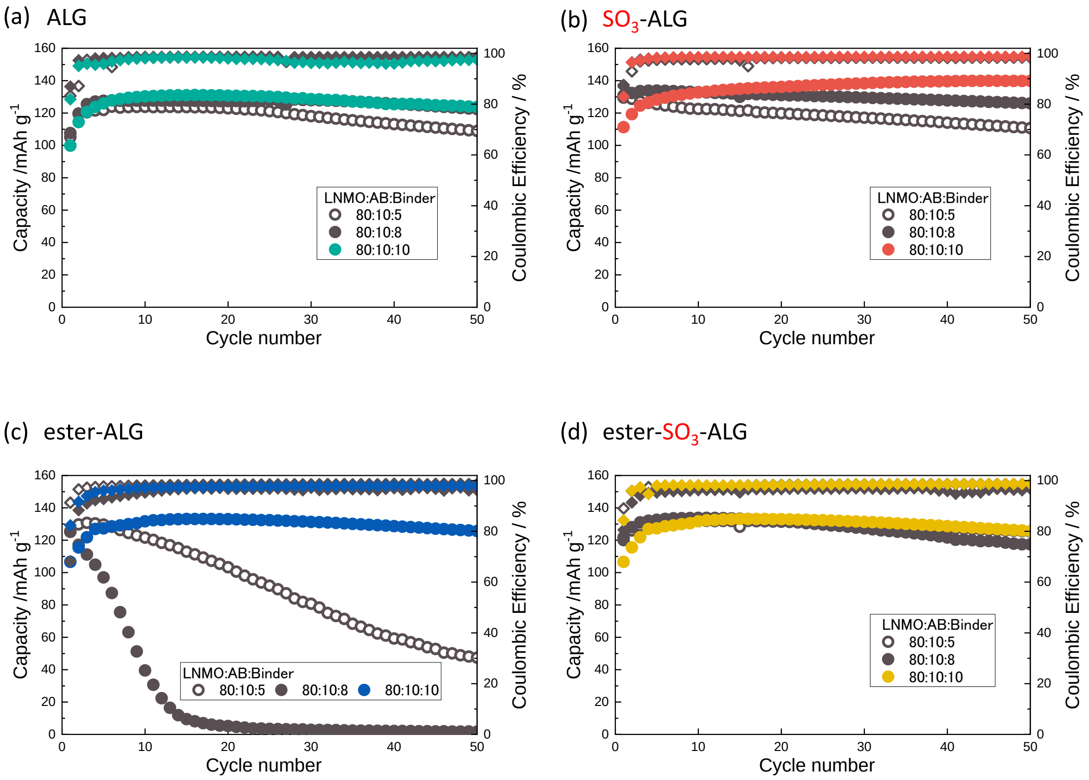

Figure S2. Variation of the capacities and Coulombic efficiencies of LNMO//Li half cells with (a) ALG, (b) SO_3-ALG, (c) ester-ALG, and (d) ester-SO_3-ALG binders, at different binder polymer contents in the composite electrodes. The cells were cycled in the voltage range of 3.5–5.0 V at 20 mA g^{-1} at 25 °C using 1 M $LiPF_6$ in EC/DMC as an electrolyte.

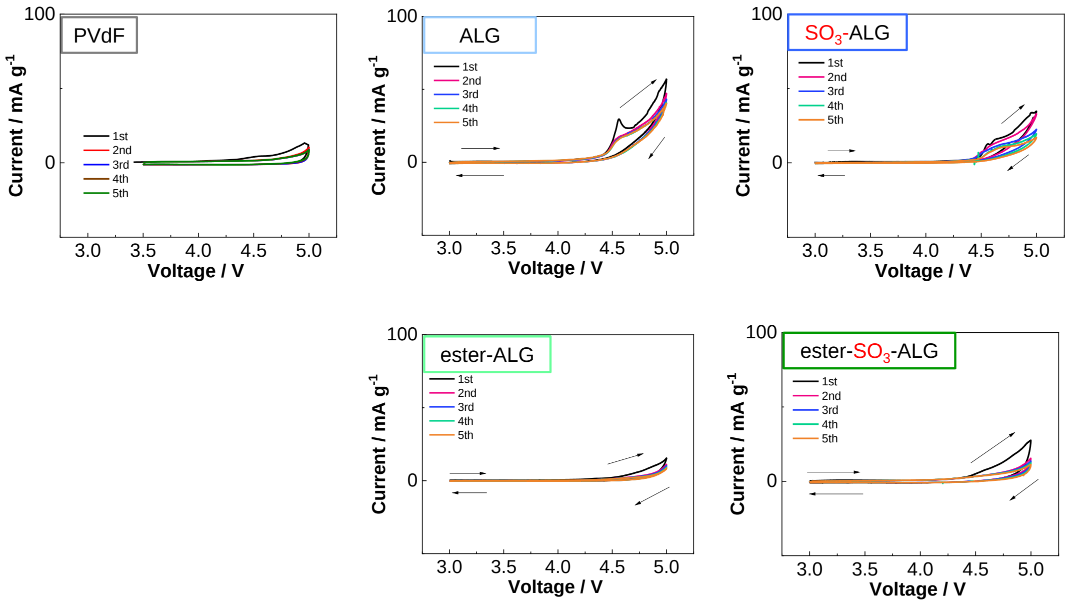

Figure S3. Cyclic voltammograms of the LNMO-free composite electrode containing 80% AB and 20% binders cycled in the voltage range of 3.0–5.0 V at 0.25 mV s^{-1} at 25 °C using 1 M $LiPF_6$ in EC/DMC as an electrolyte.

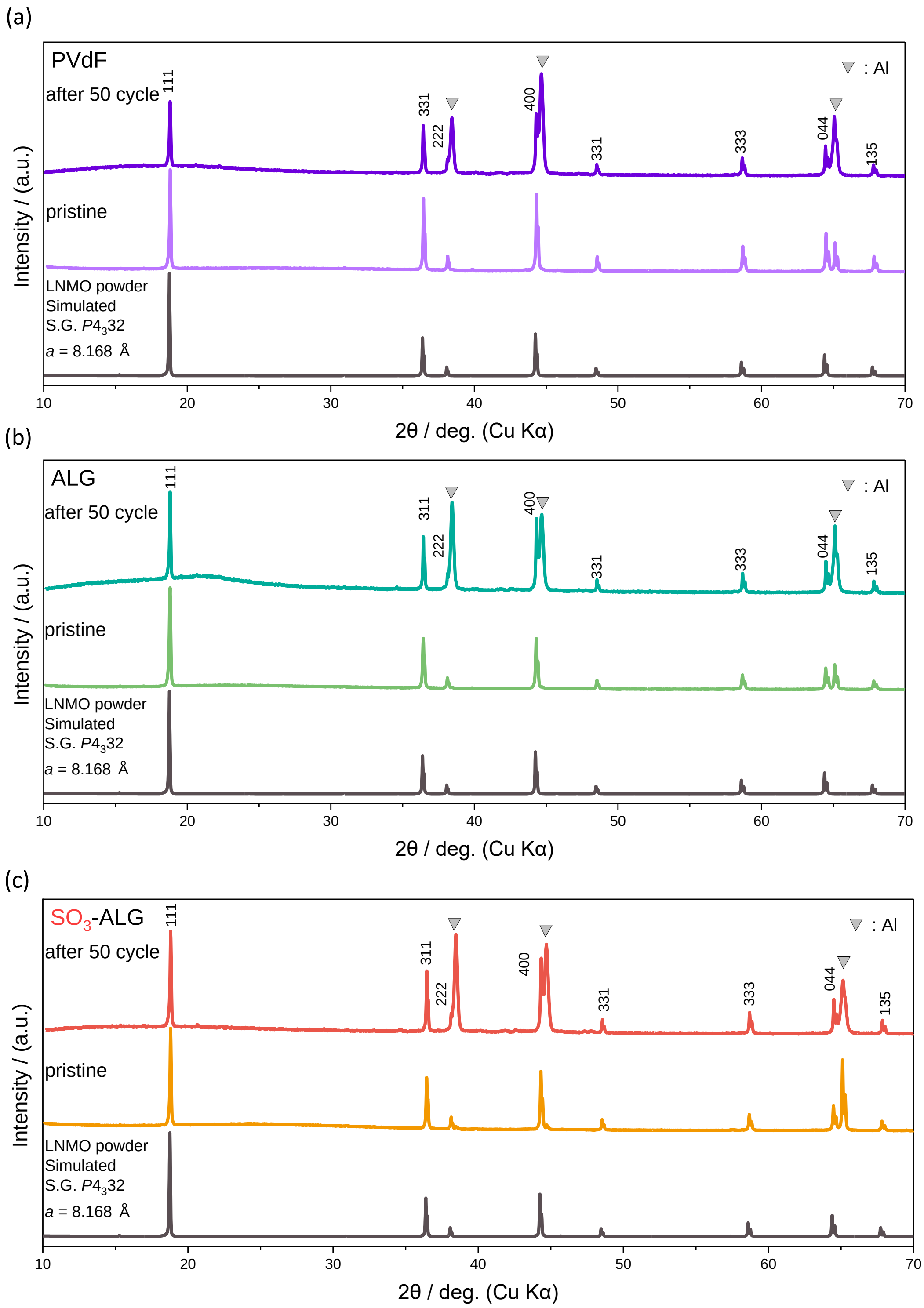

Figure S4. XRD patterns of $LiNi_{0.5}Mn_{1.5}O_4$ electrodes with (a) PVdF, (b) ALG, and (c) SO_3-ALG binders recorded before and after cycling.

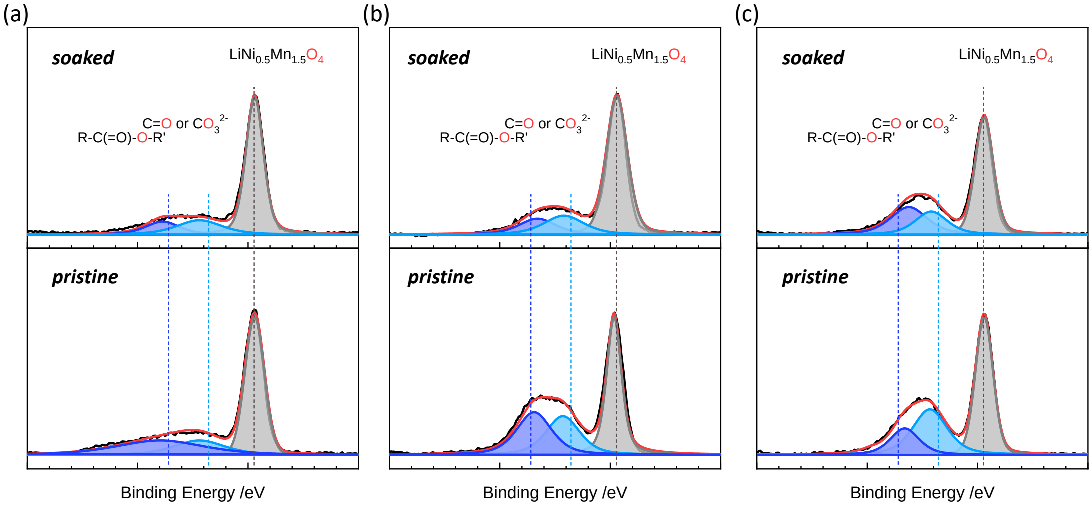

Figure S5. O 1s HAXPES profiles of pristine and electrolyte (1 M $LiPF_6$ in EC/DMC)-soaked $LiNi_{0.5}Mn_{1.5}O_4$ electrodes with (a) PVdF, (b) ALG, and (c) SO_3-ALG binders.

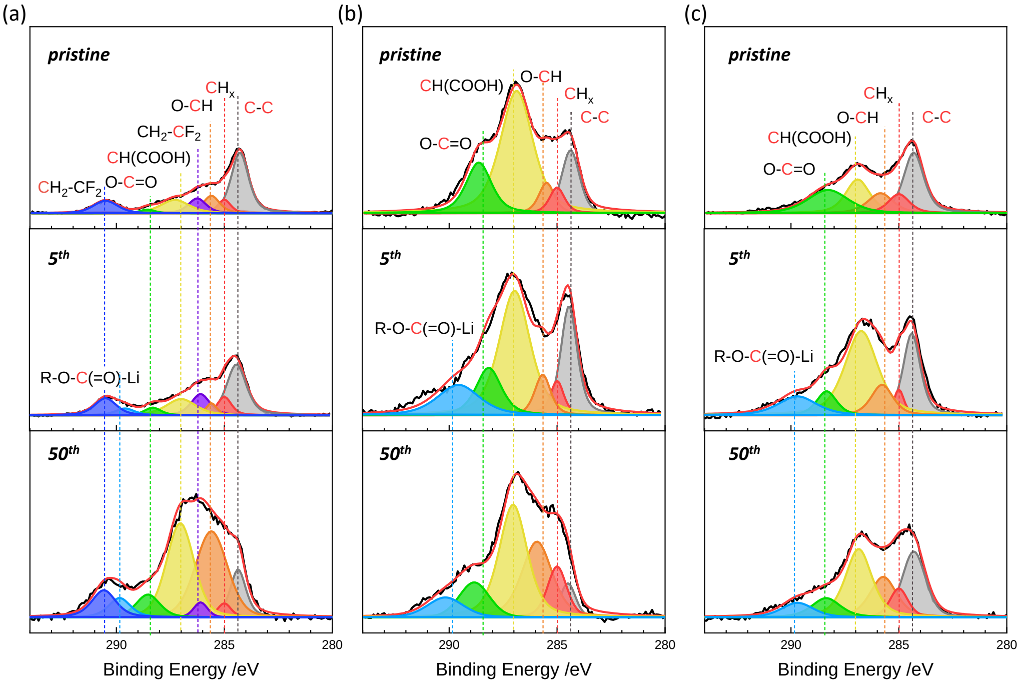

Figure S6. C 1s SOXPES profiles of $LiNi_{0.5}Mn_{1.5}O_4$ electrodes with (a) PVdF, (b) ALG, and (c) SO_3-ALG binders recorded before and after cycling.

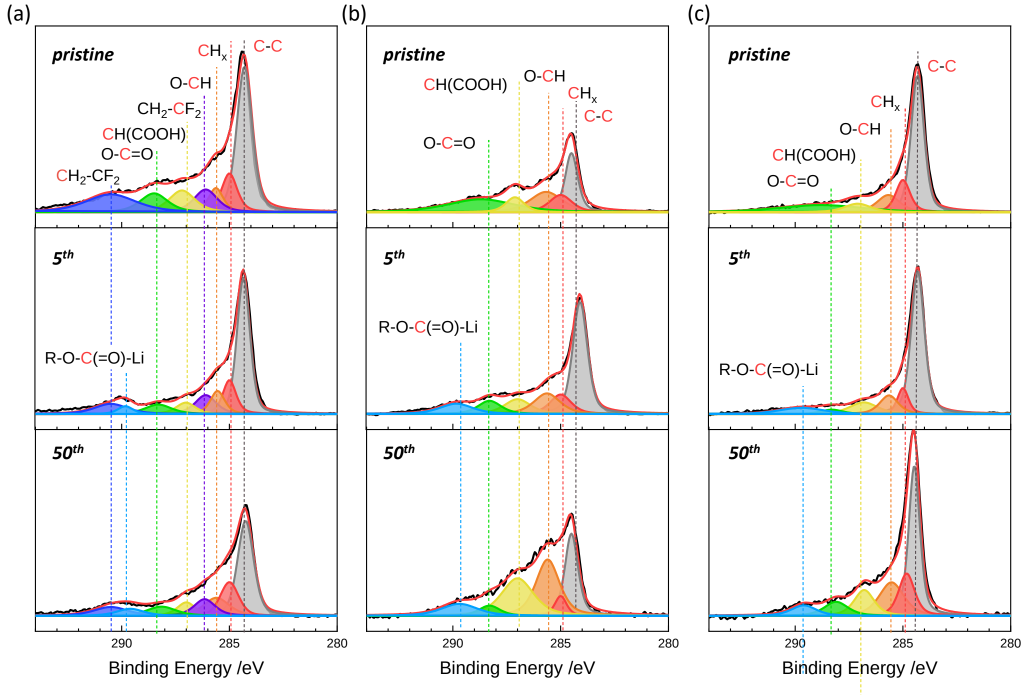

Figure S7. C 1s HAXPES profiles of $LiNi_{0.5}Mn_{1.5}O_4$ electrodes with (a) PVdF, (b) ALG, and (c) SO_3-ALG binders recorded before and after cycling.

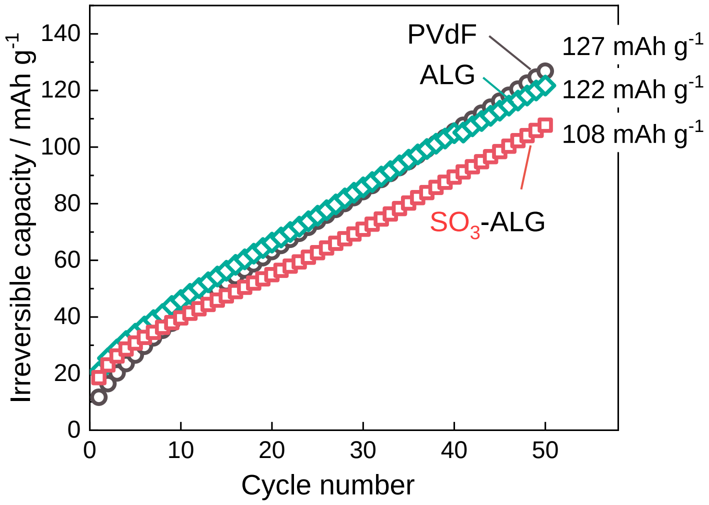

Figure S8. Effects of cycling on the irreversible capacity of $LiNi_{0.5}Mn_{1.5}O_4$//Li half cells with PVdF, ALG, and SO_3-ALG binders.

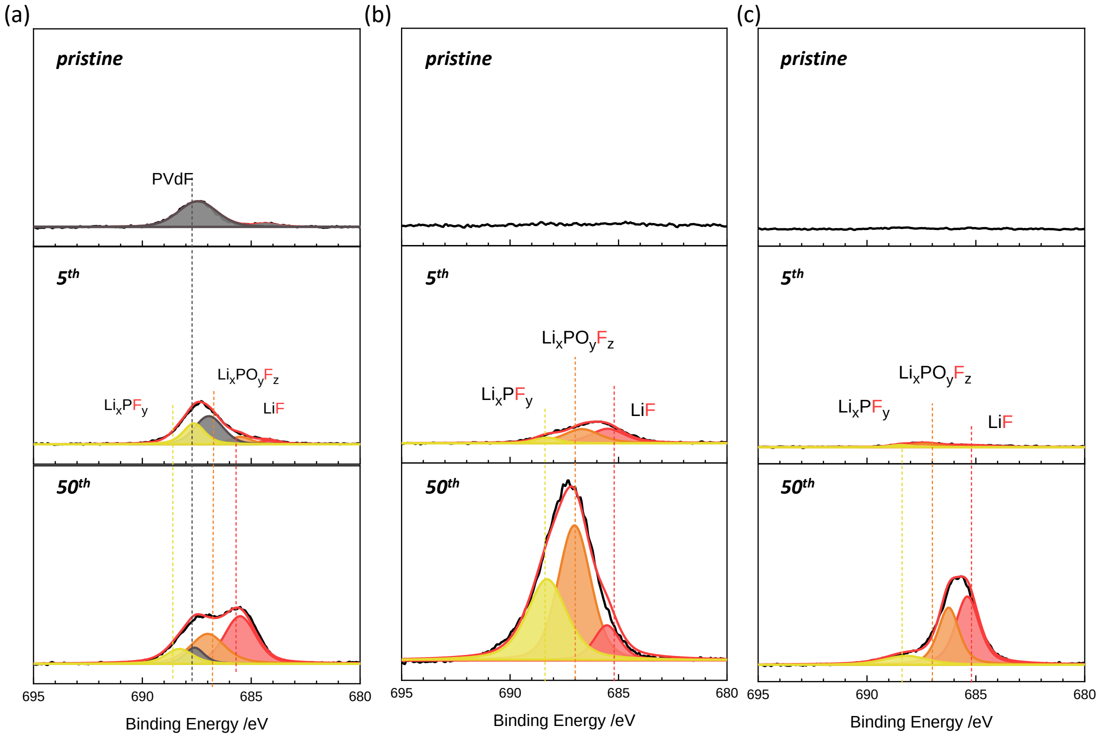

Figure S9. F 1s HAXPES profiles of $LiNi_{0.5}Mn_{1.5}O_4$ electrodes with (a) PVdF, (b) ALG, and (c) SO_3-ALG binders recorded before and after cycling.

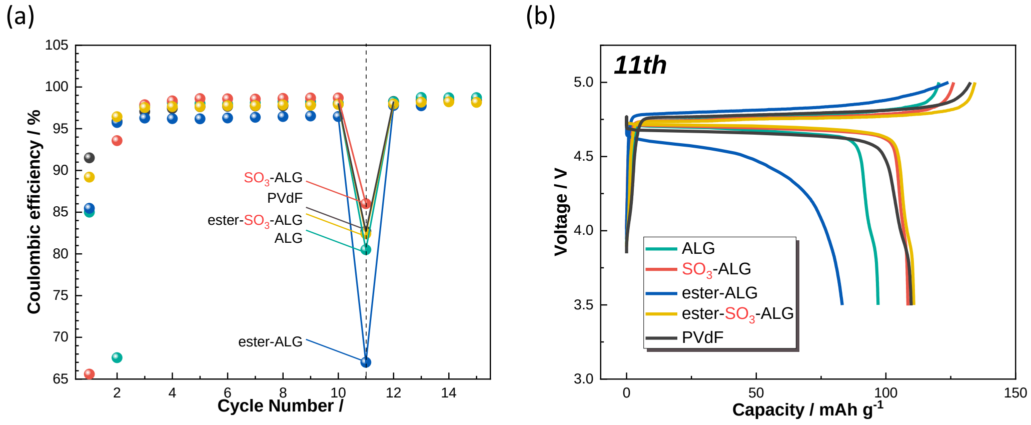

Figure S10. (a) Coulombic efficiencies and (b) eleventh-cycle charge–discharge curves obtained during the self-discharge testing of $LiNi_{0.5}Mn_{1.5}O_4$ electrodes. The cells were cycled at 20 mA g^{-1} in 1 M $LiPF_6$ in EC/DMC as an electrolyte, stored at 25°C for 7 d after the 10th charge, and cycled again.

Table S1. Eleventh-cycle coulombic efficiencies of $LiNi_{0.5}Mn_{1.5}O_4$ electrodes with various binders obtained after self-discharge.

Binder	11th coulombic efficiency (%)
PVdF	82.7
ALG	80.5
SO_3-ALG	86.0
ester-ALG	67.0
ester-SO_3-ALG	82.4

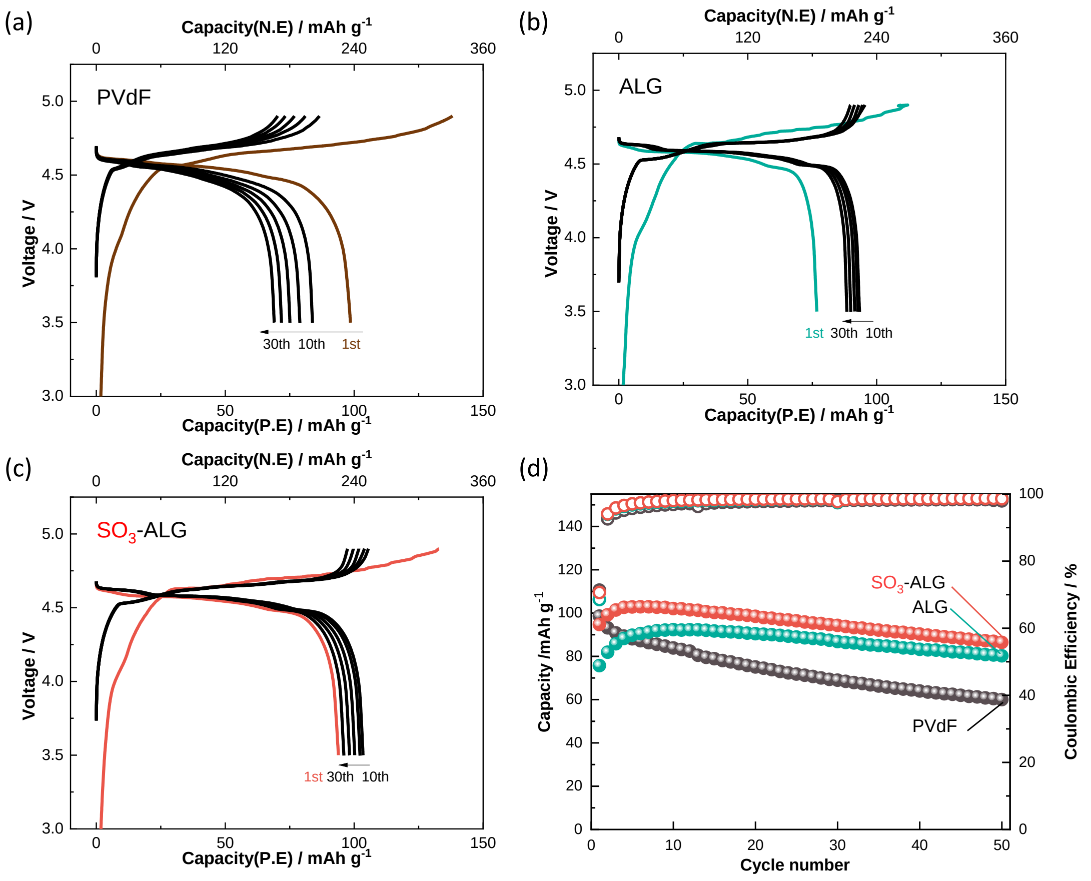

Figure S11. Charge–discharge curves of $LiNi_{0.5}Mn_{1.5}O_4$//graphite full cells with (a) PVdF, (b) ALG, and (c) SO_3-ALG. (d) Cycling performances and Coulombic efficiencies of LNMO//graphite full cells with PVdF and alginate binders. The cells were cycled at 20 mA g^{-1} in 1 M $LiPF_6$ in EC/DMC at room temperature within the voltage range of 3.5−4.9 V.

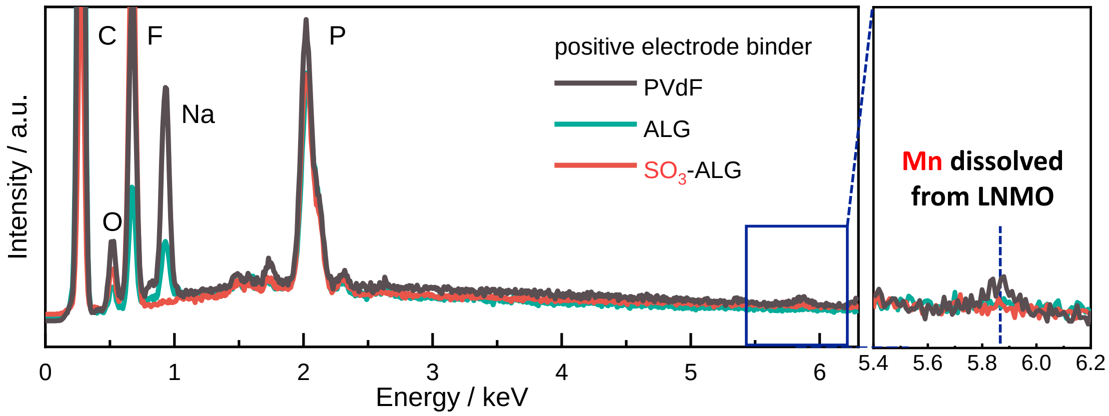

Figure S12. SEM-EDS profiles of graphite electrodes after 50 cycles in LNMO//graphite full cells.

Table S2. Surface atomic ratios estimated from the EDS profiles of graphite electrodes after 50 cycles in LNMO//graphite full cells with different binders.

Element	Positive electrode binder		
	PVdF	ALG	SO_3-ALG
C	77.1	88.8	56.6
O	1.97	0.66	1.96
F	9.95	3.63	24.2
P	10.4	6.77	17.1
Mn	0.57	0.12	0.16
Total	100	100	100
